# Роль лептина в развитии патологии эндометрия: литературный обзор

**DOI:** 10.14341/probl13397

**Published:** 2024-11-05

**Authors:** К. Д. Иевлева, И. Н. Данусевич, Л. В. Сутурина

**Affiliations:** Научный центр проблем здоровья семьи и репродукции человека; Научный центр проблем здоровья семьи и репродукции человека; Научный центр проблем здоровья семьи и репродукции человека

**Keywords:** лептин, воспаление, эндометрит, эндометриоз, имплантация эмбриона

## Abstract

Лептин является не только главным регулятором энергетического баланса в организме, но также оказывает влияние на репродуктивную и иммунную систему. Лептин и его рецепторы экспрессируются в эндометрии, активно участвуя в процессе имплантации эмбриона. По данным многочисленных исследований, изменение экспрессии и уровня лептина ассоциировано с развитием воспалительных и аутоиммунных заболеваний, в том числе эндометриоза и хронического эндометрита. Гиперпластические и воспалительные заболевания матки сопровождаются нарушением рецептивности эндометрия за счет дисрегуляции множества факторов, участвующих в процессах пролиферации, васкуляризации и децидуализации клеток. Функциональная активность большинства из этих факторов обусловлена действием лептина, однако к настоящему моменту отсутствуют исследования прямой роли лептина в патогенезе нарушений функционального состояния эндометрия при гиперпластических и воспалительных заболеваниях органов малого таза.

Таким образом, цель настоящего литературного обзора — описать предполагаемые молекулярные механизмы влияния лептина на развитие патологии эндометрия.

Литературный поиск проводился в период с 20.03.2023 по 11.05.2023 с использованием баз научной литературы: NCBI PubMed, Google Scholar (зарубежные источники), Киберленинка, Elibrary (отечественные источники), анализировались литературные источники за период 1995-2023 гг. Для поиска использовались следующие ключевые слова: лептин, эндометриальная дисфункция, эндометриальная рецептивность, воспаление, воспалительные заболевания органов малого таза.

## Abstract (en)

Leptin is not only the main regulator of energy balance, but also it affects the reproductive and immune systems. Leptin and its receptors are expressed in the endometrium and are actively involved in the embryo implantation. According to numerous studies, expression and level changes of leptin are associated with the inflammatory and autoimmune diseases, including endometriosis and chronic endometritis. Hyperplastic and inflammatory diseases of the uterus are accompanied by a violation of the receptivity of the endometrium due to the dysregulation of many factors involved in proliferation, vascularization and decidualization of cells. Activity of most of these factors is due to the leptin action, however, there are no studies of the direct effect of leptin in the pathogenesis of disorders of the endometrium in hyperplastic and inflammatory diseases.

Thus, the purpose of this literature review was to describe the putative molecular mechanisms of the effect of leptin on the development of endometrial pathology.

Literature search was carried out from 03/20/2023 to 05/11/2023 using scientific literature databases: NCBI PubMed, Google Scholar (foreign sources), Cyberleninka, Elibrary (domestic sources): references for the period 1995-2023 were analyzed. The following keywords were used for the search: leptin, endometrial dysfunction, endometrial receptivity, inflammation, pelvic inflammatory disease.

## Введение

Лептин представляет собой полипептидный гормон длинной 16 кДа, кодирующийся геном LEP и в большинстве случаев продуцируемый в жировой ткани. Главная роль лептина в организме — регуляция энергетического баланса через влияние на клеточный метаболизм и аппетит [1][2]. Рецепторы к лептину по строению гомологичны первому классу семейства цитокиновых рецепторов [3]. Существует 6 изоформ рецептора к лептину [4], но непосредственно сигнальную функцию выполняет длинная изоформа OBR1 [3], которая экспрессируется в гипоталамусе [5], а также в периферических органах и тканях [5][6]. Так, например, OBR экспрессируется на адипоцитах, иммунных клетках, в тканях яичника и эндометрия [7].

Помимо регуляции энергетического гомеостаза, лептин необходим для инициации пубертатного периода и оказывает влияние на функционирование гипоталамо-гипофизарно-яичниковой системы [8]. При этом лептин воздействует на органы и ткани не только через центральные механизмы, но и напрямую, участвуя в регуляции метаболизма, репродуктивной функции и гемопоэза [9–11]. Так, например, в жировой ткани лептин стимулирует окисление липидов и регулирует клеточный гомеостаз триглицеридов [12]. В яичниках прямое воздействие лептина стимулирует выработку стероидных гормонов, а присутствие лептина в эндометрии необходимо для нормальной реализации процессов имплантации [13][14]. Кроме того, лептин обеспечивает активность иммунной системы, стимулирует пролиферацию иммунных клеток, а также участвует в развитии воспаления [15–17].

В многочисленных исследованиях показана ассоциация уровня лептина с развитием различных хронических воспалительных заболеваний, в том числе аутоиммунных, а также с наличием заболеваний репродуктивной системы (синдром поликистозных яичников (СПКЯ), эндометриоз, хронический эндометрит), в патогенезе которых также играет роль воспаление [18–21].

Таким образом, лептин является гормоном, который играет важную роль не только в регуляции метаболизма, но также необходим для нормального функционирования иммунной системы и адекватного воспалительного ответа. Кроме того, лептин является активным участником регуляции репродуктивной функции, что позволяет предположить его роль в развитии патологии эндометрия. Целью настоящего литературного обзора явилось описать предполагаемые молекулярные механизмы влияния лептина на развитие патологии эндометрия.

Литературный поиск проводился в период с 20.03.2023 по 11.05.2023 с использованием баз научной литературы: NCBI PubMed, Google Scholar (зарубежные источники), Киберленинка, Elibrary (отечественные источники), анализировались литературные источники за период 1995–2023 гг. Для поиска использовались следующие ключевые слова: лептин, эндометриальная дисфункция, эндометриальная рецептивность, воспаление, воспалительные заболевания органов малого таза.

## Нормальный уровень лептина у женщин репродуктивного возраста

Концентрация лептина в крови человека зависит не только от количества жировой ткани, но и от пола. Так, у женщин с нормальной массой тела (18,5 ≤ индекс массы тела (ИМТ)≤ 5 кг/м2) уровень лептина в сыворотке крови составляет 23,5±1,5 нг/мл с пределами колебаний 4,7–46 нг/мл, а у мужчин с нормальной массой тела — 9±0,83 нг/мл (2,65–20,7 нг/мл) [22]. Имеющиеся различия объясняются уровнем стероидных гормонов, так как эстрогены стимулируют секрецию лептина из жировой ткани, тогда как андрогены, наоборот ингибируют [23].

В течение менструального цикла концентрация лептина изменяется в пределах от 14,9 нг/мл в раннюю фолликулярную фазу до 20,4 нг/мл в середине лютеиновой фазы с максимальным уровнем в период пика лютеинизирующего гормона (ЛГ) 21,7 нг/мл [24][25]. Имеются исследования, указывающие на синхронизацию ночной концентрации лептина с пиками ЛГ у нормально менструирующих женщин [26][27], что совпадает с результатами исследований на животных. Так, сывороточная концентрация лептина, а также уровень его экспрессии в жировой ткани, повышается в период овуляции у самок крыс [28]. Данная синхронизация концентраций лептина, ЛГ и эстрадиола лучше всего устанавливается при определении в ночные часы, что, предположительно, подтверждает гипотезу о том, что лептин регулирует колебания уровней ЛГ и эстрадиола. Максимальный уровень концентрации лептина ассоциирован с пиками концентрации эстрадиола, прогестерона, тестостерона, ЛГ при овуляции и низкими концентрациями фолликулостимулирующего гормона (ФСГ) [27][29].

Таким образом, у женщин уровень лептина выше, чем у мужчин, и зависит от фазы менструального цикла. Кроме того, уровень лептина коррелирует с концентрациями ЛГ, эстрадиола, прогестерона и тестостерона, что подтверждает его влияние на регуляцию репродуктивной функции у женщин.

## Физиологическое действие лептина на эндометрий

Впервые влияние лептина на репродуктивную функцию установили в исследованиях на мышах с делецией гена лептина (ob/ob), где выявили, что отсутствие продукции лептина обуславливало развитие у животных бесплодия, которое корректировалось введением экзогенного лептина [30]. Лептин может воздействовать на репродуктивную функцию через центральные механизмы (соматотропный релизинг-фактор) и периферические механизмы (прямое воздействие на яичники и эндометрий) [31].

Лептин и его рецепторы экспрессируются в клетках яичников и матки, при этом наибольшая экспрессия матричной рибонуклиеновой кислоты (мРНК) рецепторов к лептину обнаружена в яичниках [32], а именно в гранулезных клетках и в клетках теки [33], где они участвуют в стимуляции продукции стероидов клетками [13]. Так, лептин в низкой концентрации стимулирует, а в высокой концентрации ингибирует экспрессию ферментов, участвующих в синтезе прогестерона [34]. Также низкие дозы лептина стимулируют секрецию прогестерона, тогда как высокие концентрации, наоборот, ингибируют этот процесс [35]. Таким образом, через регуляцию выработки гормонов яичников лептин может оказывать влияние на функциональное состояние эндометрия.

Рецепторы к лептину экспрессируются непосредственно в эндометриальных клетках. Обнаружено, что снижение экспрессии данных рецепторов приводит к снижению фертильности [36] за счет их участия в регуляции процессов имплантации [37].

Уровень экспрессии лептина в эндометрии зависит от фазы менструального цикла. В исследовании эндометрия здоровых женщин репродуктивного возраста Kitawaki et al. (2000 г.) установили экспрессию рецептора к лептину, но не самого гена лептина в клетках слизистой оболочки матки. При этом наименьший уровень экспрессии лептиновых рецепторов регистрировали в середину секреторной фазы менструального цикла [36]. Однако в другом исследовании показано, что экспрессия лептина в эндометрии регистрируется только в стадию инвазии бластоцисты в эндометрий в преимплантационный период [38].

Kaplanoğlu et al. (2019 г.) провели in vitro исследование кокультивирования эмбриональных и эндометриальных клеток, по результатам которого обнаружили увеличение экспрессии рецепторов к лептину на поверхности эндометриальных клеток [39], что подтверждает необходимость присутствия лептина для реализации имплантации. В исследовании на мышах с недостаточностью лептина и бесплодием введение рекомбинантного лептина в течение восьми дней приводило к наступлению беременности. При этом беременность не наступала, если лептин прекращали вводить через 0,5–3,5 дня после спаривания, но регистрировалась, если лептин прекращали вводить через 6,5–14,5 дней после спаривания [40]. Исходя из того, что у мышей имплантация происходит на пятый день после спаривания, можно сделать вывод, что лептин необходим для этой стадии беременности [38]. Кроме того, к нарушению процессов имплантации приводило блокирование рецепторов к лептину на третий день беременности [41].

Дальнейшие исследования механизмов влияния лептина на процесс имплантации обнаружили, что лептин способен стимулировать пролиферацию и апоптоз эндометриальных эпителиальных клеток, влиять на эндометриальную рецептивность, иммунную систему матки и децидуализацию клеток эндометрия [42–44].

Так, Tanaka et al. (2008 г.) в исследовании in vitro на эндометриальных эпителиальных клетках установили, что инкубация клеточной культуры с лептином в концентрации, соответствующей физиологической норме, обуславливала пролиферацию эндометриальных клеток. Кроме того, лептин стимулировал экспрессию рецепторов к эстрогенам и прогестерону, а также функционального Fas-антигена, который является активатором клеточного апоптоза [42]. В эксперименте на человеческих эндометриальных клетках установили, что лептин влияет на децидуализацию стимулированных эпителиальных клеток эндометрия путем ингибирования секреции в них пролактина. Авторы предположили, что высокие концентрации лептина могут ингибировать апоптоз в ткани эндометрия, что в свою очередь будет тормозить децидуализацию ткани и может приводить к развитию бесплодия [43].

Yang et al. обнаружили, что лептин стимулирует экспрессию αv- и β3-интегрина в мышиных эпителиальных клетках матки, что обуславливает его действие на рецептивность эндометрия [44]. Стимулируя выработку β3-интегрина, лептин повышает адгезию бластоцисты к эндометрию [44]. Установлено, что в клетках эндометрия лептин дозозависимо стимулирует экспрессию β3-интегрина, а также матричной металлопротеиназы (MMP) 9, связывающегося с гепарином EGF-подобного фактора роста (HB-EGF) и интерлейкина (IL) 1β, остеопонтина и фактора ингибирования лейкемии (LIF), которые являются важными факторами рецептивности эндометрия [46].

Благодаря своим провоспалительным свойствам лептин также стимулирует экспрессию некоторых цитокинов: IL-6 и IL-8, регулирующего роста онкогена α (GROα), моноцитарного хемоаттрактантного белка 1 (MCP-1) и макрофагального воспалительного белка 3α (MIP3α) в эндометриальных эпителиальных и стромальных клетках [40]. Данные провоспалительные цитокины в свою очередь принимают участие в процессе имплантации эмбриона.

Таким образом, главной функцией лептина в эндометрии является регуляция процесса имплантации, которую лептин обеспечивает через влияние на факторы рецептивности эндометрия.

## Влияние лептина на иммунный ответ и развитие воспаления

Так как для лептина установлена ассоциация с развитием заболеваний, в патогенезе которых участвует воспаление [16], в том числе заболевания репродуктивной системы [19], а также нарушение иммунного ответа [21], ниже нами представлены имеющие сведения о влиянии лептина на данные процессы.

Лептин обладает плейотропным действием на клетки иммунной системы. В моноцитах и макрофагах лептин стимулирует фагоцитарную активность, пролиферацию моноцитов, оксидативный стресс, хемотаксис и индуцирование провоспалительных цитокинов (фактор некроза опухоли α (TNF-α), IL-6, IL-12), приводящие к воспалительной инфильтрации [16]. В нейтрофилах лептин оказывает стимулирующее воздействие на выработку свободных радикалов, а также поддерживает секрецию IL-1β, внутриклеточной молекулы адгезии 1 (ICAM-1) и хемокинов, которые обеспечивают процесс хемотаксиса [17]. В эозинофилах и базофилах лептин является активатором хемотаксиса, выброса цитокинов и клеточного выживания [47]. В клетках натуральных киллерах данный адипокин активирует созревание, дифференцировку, активацию и цитотоксичность, а также секрецию IL-2, IL-12, фактора роста клеток киллеров и перфорина [48].

Лептин может действовать как провоспалительный цитокин благодаря структурной схожести с IL-6. В этом случае лептин повышает экспрессию TNF-α и IL-6 [49]. Провоспалительные цитокины в свою очередь также способны регулировать уровень лептина. В исследовании in vitro на клеточной линии мышиных адипоцитов 3T3-L1 установили, что TNF-α и IL-1β зависимо от дозы и времени снижали продукцию и секрецию лептина клетками [50]. Однако в другом исследовании TNF-α снижал экспрессию лептина в клетках 3T3-L1 и жировой ткани мышей, кроме того, уровень мРНК лептина была значимо ниже у мышей с недостаточностью TNF-α в сравнении с обычными мышами [51][52]. По результатам острого эксперимента установили, что IL-1β может индуцировать повышение уровня лептина в плазме и экспрессию его гена в жировой ткани [50][53], а также в клетках эндометрия. При этом в другом исследовании на клетках 3T3-L1 инкубация с IL-1β приводила к снижению экспрессии гена лептина [54].

В исследованиях на экспериментальных моделях животных было выявлено, что лептин оказывает стимулирующее действие на развитие аутореактивности. Так, у мышей ob/ob и мышей с делецией рецептора к лептину (db/db) наблюдали резистентность к развитию индуцированных аутоиммунных заболеваний [15][55]. При этом в животной модели спонтанной аутореактивности обнаружили, что у самок мышей с нормальной массой тела и сахарным диабетом первого типа (СД1) повышенный уровень лептина предшествовал развитию заболевания. Кроме того, внутрибрюшинное введение рекомбинантного лептина животным ускоряло аутоиммунную деструкцию β-клеток поджелудочной железы, продуцирующих инсулин, и значительно повышало продукцию интерферона γ (INFγ) в периферических Т-клетках. Эти результаты показывают, что лептин может способствовать провоспалительным клеточным ответам и непосредственно влиять на развитие аутоиммунных заболеваний [56].

Роль лептина в развитии аутоиммунной патологии также подтверждена в многочисленных клинических исследованиях. Так, повышение периферической секреции лептина у человека ассоциировано с развитием таких заболеваний, имеющих в своей основе аутоиммунные механизмы, как эндометриоз, неалкогольный гепатит, хроническое воспаление легких, гломерулонефрит, синдром Бехчета, болезнь Грейвса, СД1 и ревматоидный артрит [15]. Однако результаты исследования взаимосвязи уровня лептина и перечисленных заболеваний у людей крайне противоречивы. Во многих исследованиях указывается отсутствие повышения лептина у пациентов с хроническими воспалительными аутоиммунными заболеваниями [57][58]. Интересно, что снижение потребляемых калорий или голодание приводит к снижению симптомов воспаления при некоторых аутоиммунных состояниях [59]. Однако стоит отметить, что данные улучшения могут быть связаны не только со снижением уровня вырабатываемого лептина, но и с изменениями уровня других гормонов [15].

Таким образом, основная роль лептина в регуляции иммунной системы заключается в способности повышать активность иммунитета и клеточной пролиферации и снижать апоптоз клеток киллеров. Кроме того, лептин является провоспалительным цитокином, активно участвующим в развитии воспаления, а повышение его уровня ассоциировано с развитием аутоиммунных заболеваний.

## Роль лептина в развитии воспалительных и пролиферативных заболеваний эндометрия

К настоящему моменту накоплено небольшое количество данных о роли лептина в патогенезе хронических воспалительных и гиперпластических заболеваний матки. В нашем исследовании мы установили, что у женщин с хроническим эндометритом наблюдаются более низкие концентрации сывороточного лептина. Однако это характерно для женщин без СПКЯ, тогда как у женщин с СПКЯ и хроническим эндометритом такой зависимости не прослеживается [19]. По данным Масякиной и соавт. (2015 г.), гиперлептинемия может являться фактором развития миомы матки, аденомиоза и гиперплазии эндометрия [60].

Наибольшее количество исследований, направленных на установление роли лептина в развитии воспалительных и гиперпластических заболеваний матки, связаны с изучением уровня данного гормона у пациенток с различными формами эндометриоза. Matarese et al. (2000 г.) выявили повышенный уровень лептина в сыворотке крови и в перитонеальной жидкости у женщин с эндометриозом органов малого таза по сравнению со здоровыми женщинами [21]. Также показано, что уровень лептина в перитонеальной жидкости коррелирует со стадией эндометриоза и ассоциированной с ним болью [61]. Кроме того, высокий уровень лептина наблюдается у женщин с эндометриозом, у которых могут наблюдаться проблемы с имплантацией [43].

При исследовании эктопической эндометриальной ткани выявили повышенную экспрессию лептина в ее клетках [62]. Кроме того, в исследовании с участием бесплодных женщин с эндометриомой яичника установили, что экспрессия рецептора к лептину была выше в тканях яичника с эндометриомой у бесплодных пациенток по сравнению с экспрессией рецептора к лептину в тканях нормального яичника у здоровых женщин. Также у женщин с эндометриомой наблюдали положительную корреляцию между уровнем лептина и экспрессией рецептора к нему и высокую концентрацию лептина в содержимом эндометриомы [63]. В другом исследовании у женщин с эндометриозом установили более высокий уровень сывороточного лептина по сравнению со здоровыми женщинами, при этом не вывили статистически значимых различий концентрации лептина в перитонеальной жидкости. Однако у женщин с эндометриомой яичника наблюдали более низкие значения сывороточного и перитонеального лептина в сравнении с пациентками без эндометриомы [64].

Таким образом, к настоящему моменту данные о роли лептина в развитии гиперпластических и воспалительных заболеваний матки фрагментарны и противоречивы, что может быть обусловлено как использованием различных дизайнов и методов выявления такой зависимости, так и сложностью установления механизмов влияния лептина на развитие рассматриваемой патологии.

## Возможные механизмы нарушения функционирования эндометрия, обусловленные действием лептина

В регуляции функциональной активности эндометрия ключевую роль играют маточные натуральные киллеры (uNK), которые обеспечивают процессы ангиогенеза и децидуализации в ткани эндометрия, тем самым способствуя имплантации эмбриона и нормальному течению беременности. Giuliani et al. (2014 г.) выявили, что у женщин c привычным невынашиванием беременности и бесплодием с неустановленной причиной (в том числе у женщин с эндометриозом) наблюдается повышенное содержание в эндометрии цитотоксичных uNK [65].

В норме uNK вырабатывают васкулярно-эндотелиальные факторы роста (VEGF) A и С, являющиеся главным активатором ангиогенеза в эндометрии. Кроме того, uNK вырабатывают такие провоспалительные цитокины, как IFN-γ и TNF-α, которые также способствуют ремоделированию эндометрия [66–68].

Perdu et al. (2016 г.) установили, что у женщин с ожирением по сравнению со здоровыми женщинами с нормальной массой тела наблюдается значимое снижение количества uNK в эндометрии. При этом в uNK, выделенных от женщин с ожирением, обнаружили повышенную экспрессию различных белков, связывающих инсулиноподобный фактор роста (IGFBPs) и тканевого ингибитора металлопротеиназ (TIMP), а также матричных металлопротеиназ (MMPs), которые ингибируют процессы развития трофобласта и ремоделирования сосудов [69]. Однако авторы не установили механизм снижения количества uNK в матке женщин с ожирением. Одним из возможных механизмов может быть влияние повышенной концентрации лептина, которая наблюдается при ожирении, так как лептин способен увеличивать экспрессию MMPs. Так, лептин опосредовано стимулирует экспрессию MMP-2 и 9, которые принимают активное участие в имплантации [70]. Кроме того, в NK лептин активирует созревание, дифференцировку, активацию и цитотоксичность, а также секрецию IL-2, IL-12, фактора роста клеток киллеров и перфорина [48]. Однако на настоящий момент отсутствуют данные о проявлении данных свойств лептина непосредственно на uNK.

Wu et al. (2007 г.) установили, что аномальная экспрессия лептина в эктопических эндометриальных клетках может возникать вследствие пролонгированного гипоксического стресса в перитонеальной полости под действием фактора, индуцированного гипоксией 1α (HIF-1α), который активирует промотор гена лептина [71]. Кроме того, HIF-1α стимулирует индуцированный гипоксией ангиогенез через активацию VEGF [72]. Установлено, что лептин коэкспрессируется с VEGF и стимулирует необходимые ангиогенные факторы, которые в дальнейшем повышают экспрессию VEGF. Так лептин обеспечивает процессы неоваскуляризации и модулирует ангиогенную активность VEGF в этих тканях [73][74]. Стоит отметить, что оптимальный уровень VEGFA необходим для регуляции эндометриальной рецептивности [75]. Роль лептина в развитии эндометриоза может быть объяснена его ангиогенными свойствами, однако описанные выше исследования проводились на эндотелиальных клетках сосудов, но не на клетках эндометрия.

Эндометриоз считается воспалительным заболеванием, при развитии которого наблюдают повышение уровней IL-6, IL-8, TNF-α в перитонеальной жидкости и в сыворотке крови пациенток [76][77]. Повышенная концентрация провоспалительных цитокинов наблюдается также в эндометриальной ткани женщин, страдающих хроническим эндометритом [78][79]. Наличие данных изменений может быть связано с повышенным уровнем лептина у таких пациенток, который обуславливает избыточную экспрессию и секрецию данных цитокинов [49].

Установлено, что индукцию HIF-1α в эндометриальных эпителиальных клетках может вызывать комбинация таких провоспалительных факторов, как IL-1β, TNFα и липополисахариды (ЛПС), которые принимают активное участие в патогенезе хронического эндометрита [80]. В свою очередь лептин обладает провоспалительными свойствами и повышает секрецию TNF-α, IL-6 и IL-12 [49]. Исходя из данных, что рецепторы к лептину экспрессируются в клетках эндометрия [7] и участвуют в процессе имплантации [38], в том числе за счет модулирования экспрессии провоспалительных цитокинов [46], можно предположить, что лептин способен участвовать в патогенезе развития хронического эндометрита. К настоящему моменту отсутствуют исследования, напрямую указывающие на наличие такого механизма. Предположительная схема патогенетического действия повышенного уровня лептина на рецептивность эндометрия представлена на рисунке 1.

**Figure:**
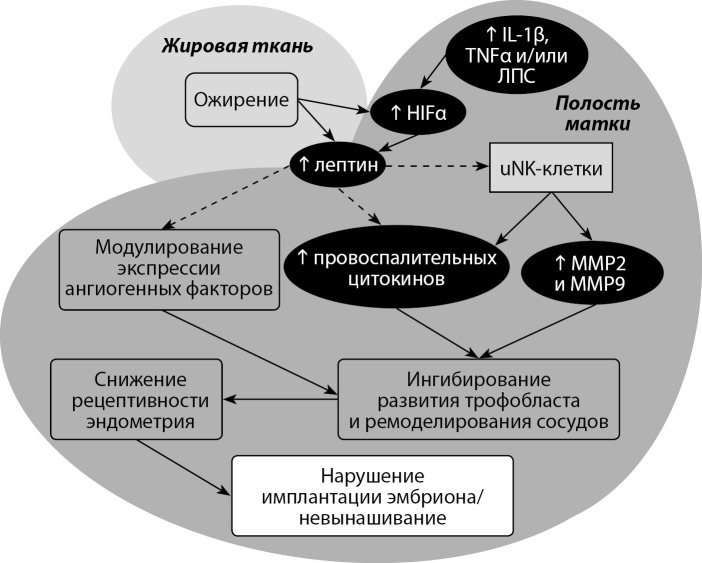
Рисунок 1. Предположительный механизм влияния повышенной концентрации лептина на рецептивность эндометрия у женщин с воспалительными и гиперплазивными заболеваниями матки. Примечание: IL-1β — интерлейкин 1β; TNFα — фактор некроза опухоли α; uNK-клетки — маточные натуральные киллеры; MMP2 — металлопротеиназа 2; MMP9 — металлопротеиназа 9; пунктирными стрелками показаны предположительные пути воздействия лептина на клетки и ткани слизистой оболочки матки.

В нашем предыдущем исследовании мы установили, что у женщин более низкая сывороточная концентрация лептина являлась протективным фактором по отношению к развитию хронического эндометрита. Однако мы не проводили исследований по установлению молекулярных механизмов выявленной зависимости [19].

Таким образом, в нарушении функции эндометрия при гиперпластических и воспалительных заболеваниях эндометрия принимают участие большое количество различных иммунных, ангиогенных и провоспалительных факторов, дисрегуляция которых может быть обусловлена различными механизмами, в том числе аномальной экспрессией, продукцией и активностью лептина. Однако в большинстве исследований прямая роль лептина в регуляции данных факторов установлена для клеток и тканей, находящихся за пределами матки, что требует проведения дополнительных исследований.

## Заключение

Лептин представляет собой гормон жировой ткани, основной функцией которого является регуляция энергетического обмена и пищевого поведения. При этом за последние десятилетия накоплены данные об его активном участии в регуляции других функций организма, в том числе репродуктивной. Рецепторы к лептину и сам лептин активно экспрессируются в тканях яичника и эндометрия [32][33], оказывая влияние на выработку стероидных гормонов [13], овуляцию и рецептивность эндометрия [14].

Нормальный уровень лептина в организме женщины зависит не только от количества жировой ткани, но и от стадии менструального цикла, что указывает на зависимость продукции лептина от уровня гипофизарных и половых гормонов [24][25]. Показано, что уровень лептина в сыворотке крови коррелирует с сывороточными концентрациями эстрадиола, прогестерона, тестостерона и ЛГ [26][27].

Уровень лептина и его рецептора в эндометрии также зависит от стадии менструального цикла [36], а его адекватная экспрессия обуславливает процесс имплантации эмбриона [38] в результате регуляции пролиферации, апоптоза и децидуализации эндометриальных клеток, а также участия в функционировании иммунной системы матки [42–44].

В многочисленных исследованиях показана ассоциация уровня лептина с развитием различных хронических воспалительных заболеваний [15], в том числе заболеваний органов малого таза (хронический эндометрит, эндометриоз) [19][21]. Это может быть обусловлено тем, что лептин за счет сродства к IL-6 обладает провоспалительными свойствами [49], а также является активным стимулятором функции лейкоцитов, в том числе натуральных киллеров [16][48].

Известно, что в регуляции функциональной активности эндометрия и имплантации эмбриона ключевую роль играют uNK. У женщин с ожирением наблюдают сниженное количество uNK в эндометрии. При этом данные клетки обладают повышенной цитотоксичностью [65]. Одним из механизмов развития данного состояния может быть повышенный уровень лептина, наблюдающийся при ожирении. В результате может происходить дисрегуляция таких важных факторов имплантации как MMPs, IGFBPs и VEGF, которые обеспечивают процессы децидуализации и неоваскуляризации эндометрия [70][73][74] а также гиперпродукция провоспалительных цитокинов IL-6, IL-12, TNF-α [49].

Несмотря на установленную роль лептина в регуляции процесса имплантации, к настоящему моменту отсутствуют исследования, подтверждающие патогенетическое значение изменений экспрессии и продукции лептина и его рецептора в развитии воспалительных и гиперпластических заболеваний эндометрия. В большинстве исследований установлена ассоциация изменения уровня лептина с наличием заболевания, но не механизмы его воздействия на развитие патологии [19][21][60]. Кроме того, отсутствуют данные о роли лептина в функционировании uNK, а также экспрессии VEGF и провоспалительных цитокинов непосредственно в эндометрии как здоровых женщин, так и женщин с воспалительными и гиперпластическими заболеваниями матки.

Таким образом, согласно имеющимся данным лептин является активным участником регуляции репродуктивной функции у женщин за счет его роли в реализации процесса имплантации эмбриона. Несмотря на установленную ассоциацию уровня лептина с наличием воспалительных и гиперпластических заболеваний эндометрия, на настоящий момент отсутствуют данные о его патогенетической роли в развитии данной патологии. Это обуславливает необходимость проведения дополнительных исследований, направленных на установление роли лептина в регуляции факторов рецептивности эндометрия как в норме, так и при патологических состояниях.

## Дополнительная информация

Источники финансирования. Работа выполнена в рамках государственной бюджетной темы №121022500180-6 «Патофизиологические механизмы и генетико-метаболические предикторы сохранения репродуктивного здоровья и долголетия в различных возрастных, гендерных и этнических группах».

Конфликт интересов. Авторы заявляют об отсутствии конфликта интересов.

Участие авторов. Иевлева К.Д. — концепция и написание рукописи; Данусевич И.Н. — концепция и редактирование рукописи; Сутурина Л.В. — концепция и редактирование рукописи. Все авторы одобрили финальную версию статьи перед публикацией, выразили согласие нести ответственность за все аспекты работы, подразумевающую надлежащее изучение и решение вопросов, связанных с точностью или добросовестностью любой части работы.
